# The Comparative Value of Eucaine and Cocaine as Anesthetics

**Published:** 1897-04

**Authors:** 


### THE COMPARATIVE VALUE OF EUCAINE AND COCAINE AS LOCAL ANESTHETICS.

The *Presse Medicale* for February 17th contains a report of a recent meeting of the Academie de Medicine at which M. Reclus presented the following results obtained from experiments which had been undertaken for the purpose of studying the action of eucaine and cocaine: 1. The injection of cocaine is not at all painful, while that of eucaine causes a certain smarting sensation. 2. Eucaine is a vaso-dilator, while cocaine is a vaso-constrictor; with the former the field of operation is clouded by the blood. 3. Eucaine is certainly an excellent analgetic, although in deep operations the perception of pain seems to be somewhat more distinct than with cocaine. 4. In an operation with cocaine, anesthesia is still complete an hour and ten minutes after the operation, while with eucaine it disappears after forty-five minutes.

If, said M. Reclus, eucaine was less toxic than cocaine, it was still to be preferred in spite of these slight inconveniences. M. Pouchet, he said, had made sixty experiments on different animals. He had recognized that the toxicity of eucaine was nearly as great as that of cocaine. He preferred the latter, which presented warning symptoms of intoxication, to eucaine, which suddenly overcame the patient without any premonitory symptoms —*New York Medical Journal.*

---

### INSTANT RELIEF OF AFTER-PAINS.

According to Winterburn (*Journal of Obstetrics*), "In many cases a nice warm meal is better than any medicine; still, where the pains are exhaustingly severe I turn to amyl nitrite. This potent drug is a very efficient controller of after-pains, and, used cautiously, I see no reason to apprehend harm from it. A neat way of using it is to saturate a small piece of tissue paper with five or six drops, stuff this into a two-drachm vial, and request the patient to draw the cork and inhale the odor when she feels the pain coming on. It acts with magical celerity."

